# Supplementary material for: Impact of Antiretroviral Therapy on Oral Health among Children Living with HIV: A Systematic Review and Meta-Analysis
Source: Int J Environ Res Public Health. 2022 Sep 21;19(19):11943. doi: 10.3390/ijerph191911943 (PMC9565507; doi:10.3390/ijerph191911943)
Supplement: Supplementary file 1 [file ijerph-19-11943-s001.zip › ijerph-1904043-supplementary.pdf]

Supplemental Material 1. Search strategies with antiretroviral therapy

PubMed
Searched on 03 Aug 2022
Results: 256

(((((HIV Infections[MeSH] OR HIV[MeSH] OR "sexually transmitted diseases, viral"[MeSH] OR AIDS[MeSH] OR hiv[tw] OR hiv-1[tw] OR hiv-2[tw] OR hiv1[tw] OR hiv2[tw] OR hiv infect*[tw] OR human immunodeficiency virus[tw] OR human immunedeficiency virus[tw] OR human immuno-deficiency virus[tw] OR human immune-deficiency virus[tw] OR ((human immun*) AND (deficiency virus[tw])) OR acquired immunodeficiency syndrome[tw] OR acquired immunedeficiency syndrome[tw] OR acquired immuno-deficiency syndrome[tw] OR acquired immune-deficiency syndrome[tw] OR ((acquired immun*) AND (deficiency syndrome[tw]))))) AND (("Adolescent"[Mesh] OR "Child"[Mesh] OR "Infant"[Mesh] OR “pediatric”[tw] OR ”paediatric”[tw] OR “adolescent”[tw] OR “teen”[tw] OR “infant”[tw]))) AND ((((((((((("Oral Health"[MeSH]) OR "Mouth Diseases"[MeSH]) OR "Oral Hygiene"[MeSH]) OR "Dental Caries"[MeSH]) OR "Periodontal Diseases"[MeSH]) OR "Gingival Diseases"[MeSH]) OR "Tooth Injuries"[MeSH]) OR "Mouth Neoplasms"[MeSH]) OR "Dentistry"[MeSH])) OR (((((((((((oral health[tw]) OR mouth disease*[tw]) OR oral hygiene[tw]) OR mouth hygiene[tw]) OR dental caries[tw]) OR periodontal disease*[tw]) OR gingival disease*[tw]) OR tooth injur*[tw]) OR mouth tumor[tw]) OR oral cancer[tw]) OR dentistry[tw]))))) AND ((("Antiretroviral Therapy, Highly Active"[Mesh] OR "Anti-Retroviral Agents"[Mesh] OR "antiretroviral"[tw] OR “ART”[tw] OR "medication"[tw])))

Medline (Ovid MEDLINE(R) 1946 to December Week 4 2019, Ovid MEDLINE(R) In-Process & Other Non-Indexed Citations 26 July 2019)

Searched on 03 Aug 2022
Results: 243

1. exp HIV Infections/ or exp HIV/ or exp sexually transmitted diseases, viral/ or exp AIDS/
2. (hiv or hiv-1 or hiv-2 or hiv1 or hiv2 or hiv infect* or human immunodeficiency virus or human immunedeficiency virus or human immuno-deficiency virus or human immune-deficiency virus).tw.
3. ((human immun* and deficiency virus) or acquired immunodeficiency syndrome or acquired immunedeficiency syndrome or acquired immuno-deficiency syndrome or acquired immune-deficiency syndrome or (acquired immun* and deficiency syndrome)).tw.
4. 1 or 2 or 3
5. exp adolescent/ or exp child/ or exp infant/
6. (pediatric or paediatric or adolescent or teen or infant).tw.

7. 5 or 6
8. exp Oral Health/ or exp Mouth Diseases/ or exp Oral Hygiene/ or exp Dental Caries/ or exp Periodontal Diseases/ or exp Gingival Diseases/ or exp Tooth Injuries/ or exp Mouth Neoplasms/ or exp Dentistry/
9. (oral health or mouth disease* or oral hygiene or mouth hygiene or dental caries or periodontal disease* or gingival disease* or tooth injur* or mouth tumor or oral cancer or dentistry).tw.
10. 8 or 9
11. 4 and 7 and 10
12. exp Antiretroviral Therapy, Highly Active/ or exp Anti-Retroviral Agents/
13. (antiretroviral or ART or medication).tw.
14. 12 or 13
15. 11 and 14

Embase (Embase Classic+Embase 1947 to 2018 December 28)

Searched on 03 Aug 2022
Results: 612

exp Human immunodeficiency virus infection/ or exp Human immunodeficiency virus/ or exp sexually transmitted disease/ or exp acquired immune deficiency syndrome/

(hiv or hiv-1 or hiv-2 or hiv1 or hiv2 or hiv infect* or human immunodeficiency virus or human immunedeficiency virus or human immuno-deficiency virus or human immune-deficiency virus).tw.

((human immun* and deficiency virus) or acquired immunodeficiency syndrome or acquired immunedeficiency syndrome or acquired immuno-deficiency syndrome or acquired immune-deficiency syndrome or (acquired immun* and deficiency syndrome)).tw.

1 or 2 or 3

exp adolescent/ or exp child/ or exp infant/

(pediatric or paediatric or adolescent or teen or infant).tw.

5 or 6

exp mouth disease/ or exp mouth hygiene/ or exp dental caries/ or exp periodontal disease/ or exp gingiva disease/ or exp tooth injury/ or exp mouth tumor/ or exp dentistry/

(oral health or mouth disease* or oral hygiene or mouth hygiene or dental caries or periodontal disease* or gingival disease* or tooth injur* or mouth tumor or oral cancer or dentistry).tw.

8 or 9

4 and 7 and 10

exp highly active antiretroviral therapy/ or exp antiretrovirus agent/

(antiretroviral or ART or medication).tw.

12 or 13

11 and 14

Scopus

Searched on 03 Aug 2022
Results: 199

(((TITLE-ABS-KEY ("HIV Infections" OR hiv OR "sexually transmitted diseases, viral" OR Aids OR "hiv-1" OR "hiv-2" OR hiv1 OR hiv2 OR "human immunodeficiency virus" OR "human immunedeficiency virus" OR "human immuno-deficiency virus" OR "human immune-deficiency virus") OR TITLE-ABS-KEY ("acquired immunodeficiency syndrome" OR "acquired immunedeficiency syndrome" OR "acquired immuno-deficiency syndrome" OR "acquired immune-deficiency syndrome")))

AND (TITLE-ABS-KEY (adolescent OR child OR infant OR pediatric OR paediatric OR teen))

AND (TITLE-ABS-KEY (" oral health" OR "Mouth Diseases" OR "Oral Hygiene" OR "Dental Caries" OR "Periodontal Diseases" OR "Gingival Diseases" OR "Tooth Injuries" OR "Mouth Neoplasms" OR "mouth tumor" OR "oral cancer" OR dentistry)))

AND (TITLE-ABS-KEY ("Antiretroviral Therapy, Highly Active" OR "Anti-Retroviral Agents" OR antiretroviral OR art OR medication))

Supplemental Material 2. Tables

Table S1. Comparison of HIV-infected individuals under antiretroviral medications versus those without medications.

	Study (Year)	Exposure	Control	Number of subjects: Exposure	Number of subjects: Control	Outcomes	OR	95% CI
Dental caries prevalence and severity								
	Ponnam (2012)	CLWH cART	CLWH NMD	95	95	**DMFT/dmft>0**	*0.91*	*(0.50,1.65)* ^{NS}
2	Oliscovicz (2015)	CLWH cART	CLWH NMD	51	14	**deft**	*NR*	*NR*
		CLWH ART	CLWH NMD	46	14	**deft**	*NR*	*NR*
				46	14	**DMFT**	*NR*	*NR*
Oral hygiene and periodontal status								
1	Ponnam (2012)	CLWH cART	CLWH NMD	95	95	**Gingivitis/ periodontitis**	1.21	*(0.68, 2.16)* ^{NS}
2	Oliscovicz (2015)	CLWH cART	CLWH NMD	51	14	**Gingivitis**	0.73	*(0.14, 3.72)* ^{NS}
		CLWH MT	CLWH NMD	46	14	**Gingivitis**	1.14	*(0.67, 1.96)* ^{NS}
	**Overall**						**0.82**	***(0.46,1.44)*** ^{NS}
	None					**Oral hygiene status**	*(Nil)*	*(Nil)*
Oral-health related WHO clinical staging 2								
1	Baghirath (2013)	CLWH ART	CLWH NMD	50	50	**Angular cheilitis**[a]	1.00	*(0.10, 9.95)* ^{NS}
2	Divarkar (2015)			62	55		0.09	*(0.00, 1.74)* ^{NS}
3	Jose (2013)	CLWH cART	CLWH NMD	47	53		0.79	*(0.15, 4.22)* ^{NS}
	**Overall**						**0.58**	***(0.17,1.99)*** ^{NS}
1	Baghirath (2013)	CLWH ART	CLWH NMD	50	50	**Linear gingival erythema**[a]	0.19	*(0.01, 4.10)* ^{NS}
2	Divarkar (2015)			62	55		0.12	*(0.01, 2.38)* ^{NS}
3	Mensana (2019)	CLWH cART	CLWH NMD	24	4		14.33	*(1.55, 132.16)* ^{NS}
4	Jose (2013)			47	53		1.15	*(0.41, 3.27)* ^{NS}
5	Oliscovicz (2015)			51	14		3.43	*(0.18, 65.81)* ^{NS}
		CLWH MT	CLWH NMD	46	14		0.96	*(0.04, 24.77)* ^{NS}
	Overall						***1.29***	**(0.58,2.88)** ^{NS}

1	Baghirath (2013)	CLWH ART	CLWH NMD	50	50	**Recurrent oral ulcerations**[a]	0.76	*(0.18, 3.26)* ^{NS}
2	Divarkar (2015)			62	55		1.09	*(0.30, 4.02)* ^{NS}
3	Jose (2013)	CLWH cART	CLWH NMD	47	53		4.04	*(1.12, 14.55)* *↑
4	Oliscovicz (2015)			51	14		0.86	*(0.03, 22.29)* ^{NS}
		CLWH MT	CLWH NMD	46	14		0.96	*(0.04, 24.90)* ^{NS}
	Overall						***1.50***	***(0.72, 3.13)*** ^{NS}
1	Oliscovicz (2015)	CLWH cART	CLWH NMD	51	14	**Persistent parotid enlargement**	0.02	*(0.00, 0.08)* *↓
		CLWH MT	CLWH NMD	46	14		0.01	*(0.00, 0.07)* *↓
	Overall						***0.01***	***(0.00, 0.05)*** *↓
Oral health-related WHO clinical staging 3								
**1**	Baghirath (2013)	CLWH ART	CLWH NMD	50	50	**Oral candidiasis**[a]	0.39	*(0.15, 1.05)* ^{NS}
**2**	Divarkar (2015)			62	55		0.36	*(0.16,0.83)**↓
**3**	Pomarico (2009)			25	15		0.17	*(0.02,1.22)* ^{NS}
	Subgroup						***0.35***	***(0.19,0.63)**↓**
**1**	Oliscovicz (2015)	CLWH MT	CLWH NMD	46	14		0.96	*(0.04,24.90)* ^{NS}
**1**	Jose (2013)	CLWH cART	CLWH NMD	47	53		0.77	*(0.33,1.76)* ^{NS}
**2**	Konstantyner (2013)			182	89		0.19	*(0.11,0.33)* *↓
**3**	Oliscovicz (2015)			51	14		0.87	*(0.03,22.48)* ^{NS}
**4**	Pomarico (2009)			24	16		0.30	*(0.05,1.64)* ^{NS}
**5**	Ponnam (2012)			95	95		0.52	*(0.26,1.03)* ^{NS}
	Subgroup						***0.35***	***(0.24,0.51)**↓**
**1**	Konstantyner (2013)	CLWH Post-cART	CLWH Pre- cART	137	284		0.33	*(0.21,0.53)* *↓
**2**	Prasitsuebsai (2012)			1480	2129		0.15	*(0.11,0.21)* *↓
**1**	Prasitsuebsai (2012)	CLWH MT/DT	CLWH Pre-MT/DT	272	2129		1.06	*(0.76,1.46)* *↓
	Overall						***0.37***	***(0.31,0.44)*** *↓
**1**	Baghirath (2013)	CLWH ART	CLWH NMD	50	50		0.13	*(0.01,2.67)* ^{NS}

**2**	Divarkar (2015)		CLWH NMD	62	55	**Oral hairy leukoplakia**[a]	0.12	*(0.01, 2.38)* NS
**3**	Jose (2013)	CLWH cART	CLWH NMD	47	44		2.75	*(0.39,19.43)* NS
	**Overall**						***0.67***	***(0.16,2.80)*** NS
**1**	Prasitsuebsai (2014)	CLWH Post-cART	CLWH Pre-ART	1480	2129	**Acute necrotizing ulcerative gingivitis/periodonitits**	0.00	*(0.00,0.03)* *↓
		CLWH MT/DT	CLWH Pre-ART	272	2129		0.00	*(0.00,0.02)* *↓
	Overall						***0.00***	***(0.00,0.02)*** *↓
Oral health-related WHO clinical staging 4								
1	Jose (2013)	CLWH cART	CLWH NMD	47	53	**Chronic herpes simplex infection**	2.75	*(0.39,19.43)* NS
2	Oliscovicz (2015)	CLWH cART	CLWH NMD	51	46		0.87	*(0.03,22.48)* NS
		CLWH MT	CLWH NMD	46	14		2.03	*(0.38,10.83)* NS
	Overall						*2.47*	*(0.88,6.97)* NS
1	Prasitsuebsai (2014)	CLWH cART	CLWH NMD	1480	2129	**Kaposi's sarcoma**[a]	0.00	*(0.00,0.03)* *↓
		CLWH MT/DT		272	2129		0.00	*(0.00,0.03)* *↓
	Overall						***0.00***	***(0.00,0.03)*** *↓
Other oral-health related diseases and conditions								
**1**	Baghirath (2013)	CLWH ART	CLWH NMD	50	50	**Hyperpigmentation**[a]	12.21	*(0.66,226.97)* NS
**2**	Divarkar (2015)	CLWH ART	CLWH NMD	62	55		0.64	*(0.22,1.92)* NS
**3**	Jose (2013)	CLWH cART	CLWH NMD	47	54		1.14	*(0.29,4.47)* NS
**4**	Ponnan (2012)	CLWH cART	CLWH NMD	95	95		8.93	*(2.30,34.60)* *↑
	Overall						***1.79***	***(0.89,3.61)*** NS
**1**	Ponnan (2012)	CLWH cART	CLWH NMD	233	55	**Mucocele**[a]	***0.02***	***(0.00,1.09)*** NS
**1**	Ponnan (2012)	CLWH cART	CLWH NMD	95	95	**Ulcerative stomatitis**	***0.03***	***(0.02,0.07)*** *↓

Legend of table:

*↑ :Significantly higher

*↓ :Significantly lower

[a] Adjusted OR was calculated with a fixed value (0.5) added to each cell in the 2x2 tables for zero-cell corrections

Note. ART= antiretroviral therapy; cART= combined antiretroviral therapy; CLWH= children living with HIV; dmft/DMFT= decayed, missing, filled teeth for primary/permanent dentition; DMFS/dmfs = decayed, missing, filled tooth surface for primary/permanent dentition; DT= dual-therapy; CLWH, HIV; MD= Under medications; MT= Monotherapy; NR= Not reported; NRTI= nucleoside reverse transcriptase inhibitor; NNRTI = non-nucleoside reverse transcriptase inhibitors; NMD= No medication; OR = odds ratio; PI= protease inhibitors; SMD = standardized mean difference; 95% CI = 95% confidence interval.

Table S2. Comparison of HIV-infected individuals under different antiretroviral medications.

	Study (Year)			Number of subjects		Outcomes		
		Exposure	*Control*	*Exposure*	*Control*			
Dental caries prevalence and severity								
							Mean (SD)	
1	Oliscovicz (2015)	CLWH cART	CLWH ART	51	46	**DMFT**	*cART: 3.2 (NR)*<br>*ART: 2.8 (NR)*	
		CLWH cART	CLWH ART	51	46	**dmft**	*cART: 1.9 (NR)*<br>*ART: 1.6 (NR)*	
2	Birungi (2020)	CLWH PI	CLWH<br>NRTI	80	84	**DMFT/dmft**	*PI: 1.7 (2.4)*<br>*NRTI: 2.3 (2.7)*	
		Exposure	*Control*	*Exposure*	*Control*		***OR***	***95% CI***
1	Birungi (2020)	CLWH PI	CLWH<br>NRTI	80	84	**DMFT/dmft**	*0.98*	*(0.57, 1.67)* ^{NS}
Oral hygiene and periodontal status								
1	Oliscovicz (2015)	CLWH cART	CLWH ART	51	46	**Gingivitis**	*2.00*	*(0.63,6.36)* ^{NS}
Oral health-related WHO clinical staging 2								
	None					**Angular cheilitis**	*(Nil)*	*(Nil)*
1	Oliscovicz (2015)	CLWH MT	CLWH NMD	51	46	**Linear gingival erythema**[a]	0.22	*(0.03,1.97)* ^{NS}
1	Oliscovicz (2015)	CLWH cART	CLWH MT	51	46	**Recurrent oral ulcerations**	1.11	*(0.07,18.24)* ^{NS}
1	Oliscovicz (2015)	CLWH cART	CLWH MT	51	46	**Persistent parotid enlargement**[a]	0.06	*(0.01,0.28)* *↓
Oral health-related WHO clinical staging 3								
**1**	Fangan (2000)	CLWH cART	CLWH RTI	24	25	**Oral candidiasis**	*0.38*	*(0.08,1.76)* ^{NS}
**2**	Konstantyner (2013)	CLWH cART	CLWH MT	182	82		*0.46*	*(0.26,0.83)* *↓
**3**	Konstantyner (2013)	CLWH cART	CLWH DT	182	66		*0.15*	*(0.08,0.29)* *↓
**4**	Oliscovicz (2015)	CLWH cART	CLWH MT	51	46		*0.90*	*(0.05,14.83)* ^{NS}
**5**	Prasitsuebsai (2012)	CLWH cART	CLWH MT/DT	1480	272		*0.15*	*(0.10,0.22)* *↓
	**Overall**						***0.22***	***(0.16,0.29)*** *↓

	None					**Recurrent oral candidiasis**	*(Nil)*	*(Nil)*
	None					**Pseduomembranous candidiasis**	*(Nil)*	*(Nil)*
	None					**Erythematous candidiasis**	*(Nil)*	*(Nil)*
	None					**Oral hairy leukoplakia**	*(Nil)*	*(Nil)*
**1**	Prasitsuebsai (2014)	CLWH Post-cART	CLWH MT/DT	1480	272	**Acute necrotizing ulcerative gingivitis/periodonitits**	*0.06*	*(0.00,1.50)[NS]*
Oral health-related WHO clinical staging 4								
1	Oliscovicz (2015)	CLWH cART	CLWH MT	51	46	**Chronic herpes simplex infection**[a]	0.01	(0.00,0.20) *↓
2	Prasitsuebsai (2014)	CLWH cART	CLWH MT	1480	272	**Kaposi's sarcoma**[a]	0.00	(0.00,0.22) *↓
Other oral health-related diseases and conditions								
	None					**Hyperpigmentation**	*(Nil)*	*(Nil)*
	None					**Mucocele**	*(Nil)*	*(Nil)*
	None					**Ulcerative stomatitis**	*(Nil)*	*(Nil)*

Legend of table:
*↑ :Significantly higher
*↓ :Significantly lower
[a] Adjusted OR was calculated with a fixed value (0.5) added to each cell in the 2x2 tables for zero-cell corrections

Note. ART= antiretroviral medication; cART= combined antiretroviral therapy; CLWH= children living with HIV dmft/DMFT= decayed, missing, filled teeth for primary/permanent dentition; DMFS/dmfs = decayed, missing, filled tooth surface for primary/permanent dentition; DT= dual-therapy; MD= Under medications; MT= Monotherapy; NR= Not reported; NRTI= nucleoside reverse transcriptase inhibitor; NNRTI = non-nucleoside reverse transcriptase inhibitors; NMD= No medication; OR = odds ratio; PI= protease inhibitors; RTI= reverse transcriptase inhibitors ; SMD = standardized mean difference; 95% CI = 95% confidence interval.

Supplemental Material 3. Figures

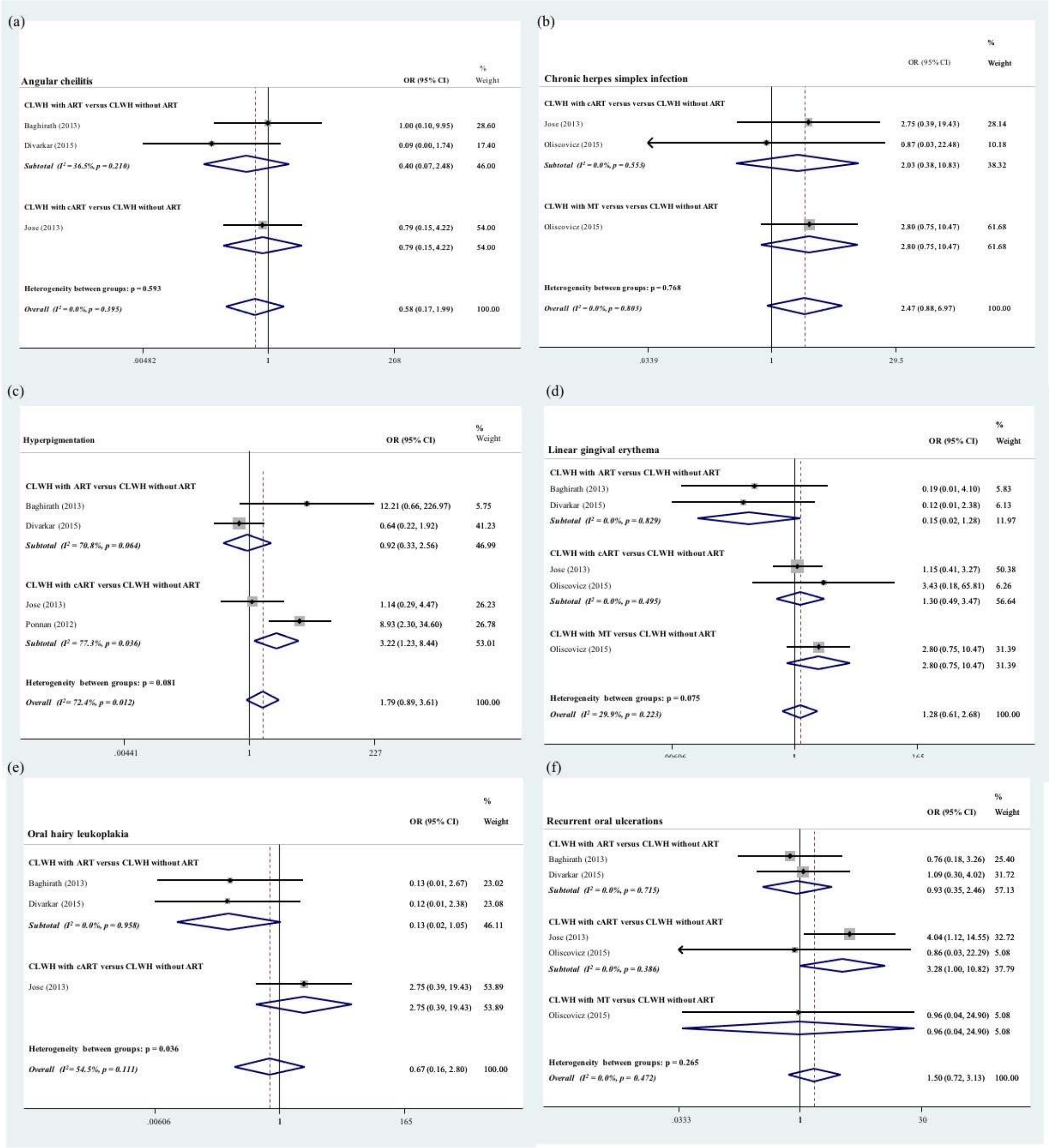

Figure S1. Prevalence of (a) angular cheilitis (b) chronic herpes simplex infection (c) hyperpigmentation (d) linear gingival erythema (e) oral hairy leukoplakia (f) recurrent oral ulcerations between CLWH with and without ART.

Note. ART= antiretroviral medication; cART= combined antiretroviral therapy; CLWH=children living with HIV; DT= dual-therapy; MT= Monotherapy; OR = odds ratio; 95% CI = 95% confidence interval
